# Cell-Type Specific Variation in X-Chromosome Dosage Compensation in Drosophila

**DOI:** 10.17912/micropub.biology.001501

**Published:** 2025-02-25

**Authors:** Soumitra Pal, Brian Oliver, Teresa M. Przytycka

**Affiliations:** 1 Neurobiology Neurodegeneration and Repair Lab, National Eye Institute, National Institutes of Health, Bethesda, Maryland, United States; 2 Intramural Research Program, National Library of Medicine, National Institutes of Health, Bethesda, Maryland, United States; 3 O’Neill School of Public and Environmental Affairs, Indiana University, Bloomington, Indiana, United States; 4 National Institute of Diabetes and Digestive and Kidney Diseases, National Institutes of Health, Bethesda, Maryland, United States

## Abstract

Male *Drosophila melanogaster* require dosage compensation to equalize X-linked gene expression with autosomal expression. Leveraging the single-nucleus Fly Cell Atlas (FCA) dataset, which includes 388,918 nuclei across diverse tissues, we investigated cell-type-specific patterns of X-chromosome dosage compensation. Our analysis identified a continuum of cell groups based on their X-to-autosome (X/A) expression ratios ranging from anti-compensated to effectively compensated and overcompensated. Anti-compensation was predominantly observed in male reproductive tissues, while overcompensation was prevalent in neural cells. The expression levels of the dosage compensation machinery's non-coding RNAs, *RoX1* and *RoX2* , correlated with compensation levels, but were insufficient to fully explain the observed patterns of compensation. These findings reveal the complexity of dosage compensation and suggest that its regulation by the *RoX* RNAs is nonlinear, implicating potential alternative mechanisms in certain cell types.

**Figure 1. Cell Type-Specific Dosage Compensation Revealed by the Fly Single Cell Atlas:**
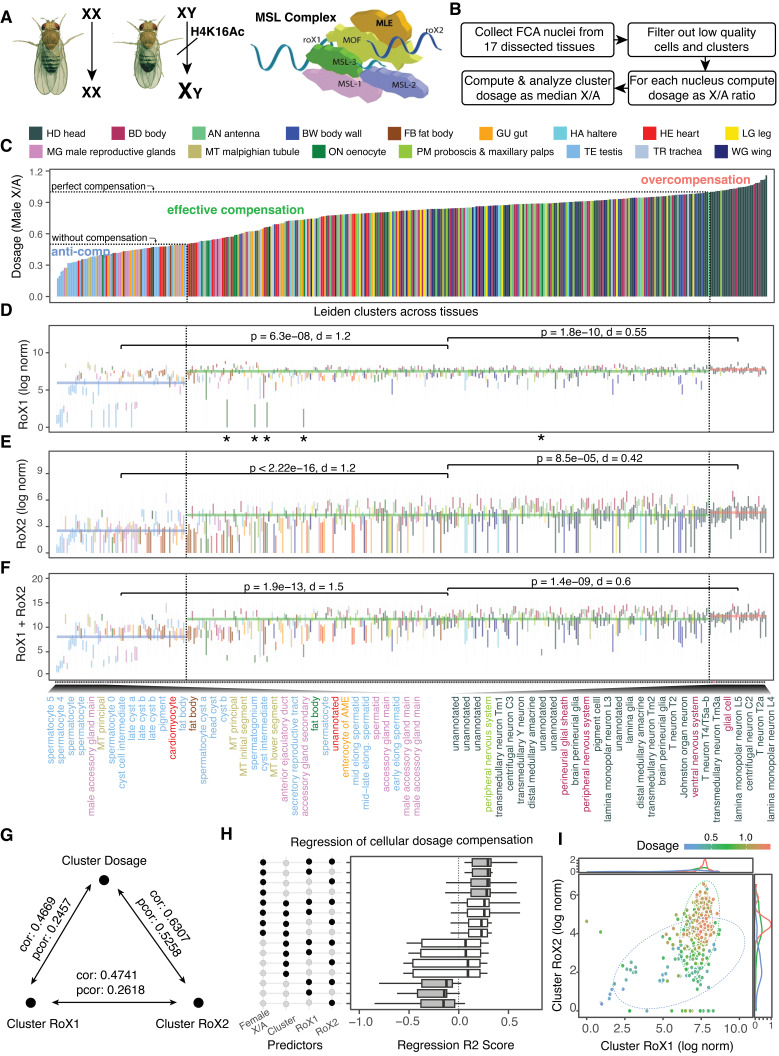
(A) In *Drosophila melanogaster* , dosage compensation is achieved by upregulating transcription from the single male X chromosome to equalize X-linked gene expression with females, who have two X chromosomes (adapted cartoon from Mendjan & Akhtar, 2007). This process is mediated by the Male-Specific Lethal (MSL) complex, a multi-protein assembly that includes MSL1, MSL2, MSL3, MOF (Male absent on the first; a histone acetyltransferase), and MLE (Maleless), along with two noncoding RNAs on X, *RoX1* and *RoX2* , that serve as scaffolds and guide the MSL complex to high-affinity binding sites (HAS) on the male X chromosome. The MOF protein within the complex acetylates histone H4 at lysine 16 (H4K16ac), loosening chromatin structure and enhancing transcriptional activity of X-linked genes. This results in a two-fold increase in transcription, balancing gene expression between the sexes. In females, the formation of the MSL complex is prevented by repression of MSL2 by the Sex-lethal (SXL) protein, ensuring male-specific dosage compensation. (B) Workflow for analyzing data from the Fly Cell Atlas (FCA). (C-F) Cluster-level statistics across 409 clusters (considering only male cells), all sharing the same x-axis. Due to space constraints, only the 30 leftmost and 30 rightmost cluster labels are displayed on the x-axis of the bottom-most plot. (C) Dosage compensation level for each cluster, with bars colored by tissue type as indicated in the legend (top). (D-F) Box plots showing cluster-specific *RoX* metrics: expression levels of *RoX1* (D) and *RoX2* (E), their sum (F). Horizontal line segments indicate the median value for each *RoX* metric across all cells within each dosage compensation category, with statistical significance assessed using a t-test and effect size computed using Cohen's d function, both using the cluster median values. Each box plot (D-F) displays the first and third quartiles as the hinges, with the median represented by the central line. The upper whisker extends to the largest value within 1.5 times the interquartile range (IQR) from the upper hinge, while the lower whisker extends to the smallest value within 1.5 times the IQR from the lower hinge. The IQR is defined as the distance between the first and third quartiles. The asterisks below the boxplots in D represent non-gonad clusters that have log-normalized *RoX1* expression levels less than 5. (G) Pearson partial and total correlations among cluster-level *RoX1* and *RoX2* expressions, and dosage compensation levels. (H) Box plot of adjusted R-squared values for regression models predicting dosage compensation in individual male cells, based on combinations of four independent variables: *RoX1* and *RoX2* expression, cluster label, and a cluster-specific baseline dosage level determined by the X/A ratio of female cells in the cluster. Each boxplot represents results from 10-fold cross-validation. Gray boxes correspond to linear regression, while white boxes represent Ridge regression. (I) Scatter plot of cluster-level *RoX1* and *RoX2* expression. Points are colored by dosage level, and ellipses group the points into three dosage categories: anti- (blue), effective (green), and over (red) compensation. Marginal distributions for *RoX1* and *RoX2* expression, separately for each category, are displayed along the top and right axes, respectively.

## Description

Female Drosophila possess two X chromosomes, while males have only one, resulting in an aneuploid condition for males (Kuroda et al., 2016). To compensate for this dosage imbalance, males employ a complex composed of several proteins collectively known as Male Specific Lethals (MSLs), along with one or both of the two non-coding RNAs encoded on the X chromosome ( *RoX1* and *RoX2* , **Fig. 1A** ). These components work together to upregulate expression of the male X chromosome (Kuroda et al., 2016). However, it remains uncertain whether all male somatic cells undergo dosage compensation and, if so, whether this process universally involves the same mechanism (Lee & Oliver, 2018). Advances in single-cell genomics now allow for the investigation of X-chromosome dosage compensation across different male cell types.

The single-nucleus Fly Cell Atlas (FCA) dataset (Li et al., 2022) comprises expression profiles from 385,099 nuclei (post-filtering) derived from 15 distinct, sexed tissue dissections, as well as replicated samples from sexed bodies and heads ( **Fig. 1B, Extended Data Table 1, Methods** ). For robust analysis, we selected Leiden resolution levels (Traag et al., 2019; Li et al., 2022) to obtain clusters with enough nuclei for analysis. Specifically, we utilized clusters at Leiden resolution of 4.0 for larger datasets (body and head) and 1.0 for dissected tissues. Notably, our findings remained consistent across resolution levels. Clusters containing fewer than 100 male nuclei were excluded, resulting in 409 distinct cell-type clusters. Ranking the clusters by median X/A ratio of their male cells revealed a range of compensation levels, which we placed into three distinct groups: 75 clusters with dosage anti-compensation (<0.5), 301 clusters with effective dosage compensation (0.5 to 1.0), and 33 clusters with X-chromosome over-expression (>1.0; **Fig. 1C** ).

The clusters exhibiting X-chromosome overcompensation were predominantly head cell types (28 of 33 clusters; 85%). These clusters were notably enriched in two cell classes: nervous system cells, including sensory cells (26; 79%), and epithelial cells (7; 21%). The 75 clusters with the dosage anti-compensation were enriched in clusters from male reproductive tissues including the testis (30/75; 40%) and reproductive glands (14/75; 19%). Anti-compensation in germ cells was expected, as X-chromosome inactivation occurs in spermatocytes (Lifschytz & Lindsley, 1972; Mahadevaraju et al., 2021), and the MSL complex does not associate with the X chromosome in male germ cells (Rastelli & Kuroda, 1998). More surprisingly, several somatic cell types in the testis, such as somatic cyst cells surrounding germ cell clusters and pigment cells of the testis sheath, also showed anti-compensation. This suggests that many cell types with male-specific functions may not be subject to typical X chromosome regulation, including dosage compensation and X inactivation, and/or may have evolved through the relocation of male-related genes from the X chromosome to autosomes.

Mechanistically, dosage compensation may correlate with the expression levels of the dosage compensation machinery. To investigate whether this machinery is less expressed in cell types with anti-compensation and more expressed in those with strong or overcompensation, we analyzed the expression of key components across various cell types. While the components of the MSL complex are not exclusively transcribed in males (Kuroda et al., 2016), complicating the analysis of nascent and nuclear transcripts in the FCA (Li et al., 2022), the non-coding *RoX* genes are highly expressed and exhibit a characteristic expression pattern that marks somatic male cell identity (Li et al., 2022). Subtle variations in *RoX* expression were evident across different FCA cell types ( **Fig. 1C- E** ).

We observed statistically significant differences in the expression of *RoX1* and *RoX2* across the three dosage compensation groups. However, the effect size (Cohen's d) for the difference between clusters with effective dosage compensation (~2-fold) and those with overcompensation was small (d = 0.55 for *RoX1* and 0.42 for *RoX2* , **Fig. 1D, E** ). In contrast, the effect size for the difference between anti-compensated group was larger (d = 1.2 for both *RoX1* and *RoX2* , **Fig. 1D, E** ). Notably, male reproductive cell types with anti-compensation exhibited median *RoX* expression levels lower than half of those in the effectively compensated cell types. This observation suggests that reduced levels of dosage compensation machinery may underlie the anti-compensation observed in these cells.

Although *RoX1* and *RoX2* are partially interchangeable — as males missing one of them remain viable, while deletion of both is lethal (Meller & Rattner, 2002) — complete functional redundancy is rare in nature. Evolutionary pressures often lead to neofunctionalization, subfunctionalization, or gene degradation (Lynch & Conery, 2000). Consistent with this, we observed notable differences in the expression patterns of *RoX1* and *RoX2* . Specifically, *RoX2* exhibited greater variation in expression than *RoX1* across cell types from all three groups ( **Fig. 1E** ), whereas the variation in total *RoX* expression ( *RoX1* + *RoX2* ) was intermediate ( **Fig. 1F** ). This suggests that *RoX1* may play a universal role in dosage compensation across cells while *RoX2* contributes to cell type-specific compensation ( **Fig. 1D- F** ). Interestingly, *RoX2* showed a stronger correlation with dosage compensation levels than *RoX1* , suggesting that it has a stronger compensation effect ( **Fig. 1G** ). However, the partial correlation of either *RoX* gene's expression with dosage compensation, while controlling for the expression of other *Rox* gene, remained high ( **Fig. 1G** ), indicating unique contributions from each of the *RoX* genes. While either *RoX1* and *RoX2* are essential for male dosage compensation, their retention does not appear to be solely driven by identical dosage-compensation requirements. This implies potential functional divergence between the two, with *RoX2* potentially having a more specialized role in fine-tuning dosage compensation.

We also identified exceptional cell type clusters that exhibited compensation similar to other cell clusters in the same compensation group but had notably low *RoX1* expression or lower *RoX2* / *RoX1* ratios. This variability may be attributed to factors such as individual genes responding differently to dosage compensation machinery (Straub & Becker, 2007). Additionally, some cell types may utilize alternative dosage compensation mechanisms, such as pairing-dependent silencing (Lee & Oliver, 2018) or differential polyploidization. However, most of these exceptions were observed in cell types specific to the male gonad. Among clusters with *RoX1* expression levels below 5, only five were found outside the male gonads (marked with asterisks in **Fig. 1D** ). Interestingly, all off these five clusters are from oenocytes and they showed higher *RoX2* expression, which appeared to compensate for the lower *RoX1* level ( **Extended Data Table 1** ).

Finally, we evaluated the effectiveness of predicting dosage compensation using *RoX1* and *RoX2* expression levels, cluster labels, and a baseline dosage level derived from female cells. Specifically, we compared adjusted R-squared values from linear regressions on various combinations of these predictors and Ridge regressions when the discrete factor cluster label was involved ( **Fig. 1H** ). Models that included baseline dosage consistently yielded better predictions, while the addition of *RoX* expressions provided only minimal improvement beyond models using cluster labels and baseline dosage levels. This indicates a potential nonlinear role of *RoX* expressions in determining dosage compensation. Supporting this hypothesis, the scatter plot of cluster medians of *RoX1* and *RoX2* expression ( **Fig. 1I** ) reveals that overcompensated clusters align with extreme *RoX1* values but not necessarily with extreme *RoX2* values. This pattern suggests that *RoX1* plays a crucial role in overcompensated clusters, while *RoX2* may function as a secondary modulator of dosage compensation.

In conclusion, dosage compensation exhibits greater variability between cell types than previously expected based on the tightly regulated dosage compensation observed at the organism level. However, it does correlate with *RoX* expression levels. Although dosage compensation is a chromosome-wide mechanism, individual genes may respond variably to it (Straub & Becker, 2007), much like how they respond to chromosomal anomalies on autosomes due to standard gene regulation (Zhang et al., 2010; Lee et al., 2016). In other words, while the median dosage compensation may approach two-fold, individual genes can exhibit a distribution of compensation with two-fold as the mean. The responses of individual genes are certainly variable across cell types, and some cell types may predominantly express genes that do not undergo fine-tuned dosage compensation. For instance, X-linked genes under strong male-specific regulation in male reproductive tissues may not require dosage compensation. Genes that are upregulated by several orders of magnitude due to specific regulatory mechanisms might not need the two-fold fine-tuning provided by dosage compensation. Additionally, it appears that evolutionary pressures have shifted highly expressed male-biased genes to autosomes, resulting in fewer X-linked genes being expressed in male cell types (Betrán et al., 2004; Sturgill et al., 2007), although this is thought to be restricted to germline expression. This alternative model suggests that the reduced number of X-linked genes expressed in certain male somatic cell types could contribute to the observed overall lower expression of the X chromosome. These ideas, while emerging from the data, will require further follow-up analysis and targeted experiments for refined hypothesis generation.

## Methods

**Datasets** : Loom files for each of the 17 tissue datasets, comprising a total of 483,286 nuclei, were downloaded from the FCA website (https://flycellatlas.org). Detailed information about these datasets is provided in the FCA_Samples sheet of **Extended Data Table 1** . The loom files were converted to AnnData format using the loompy v3.0.7 (https://linnarssonlab.org/loompy) and anndata v0.10.9 Python packages.

**Cell Filtering:** For the dosage compensation analysis, we excluded cells with likely artifacts. Specifically, male cells with zero total reads (UMI count) for *RoX1* and *RoX2* , and female cells with non-zero reads for either *RoX* gene, were removed.

**Clustering Resolution:** To address potential biases arising from the high number of unannotated nuclei in the FCA dataset, we employed Leiden clustering instead of relying solely on FCA-provided annotated cell types. For comprehensive analyses, Leiden clustering was applied at resolution 4.0 for the larger datasets (body and head) and resolution 1.0 for the dissected tissues. Clustering resolutions were selected based on manual inspection of the resulting number of clusters at each resolution and the distribution of male and female nuclei within each cluster (see LeidenClustersSummary in **Extended Data Table 1** ). The chosen resolutions ensured that the number of clusters was greater than the number of annotated clusters while avoiding clusters with very few cells. We annotated each of these Leiden clusters based on the predominant annotation of the cells within the cluster. These annotations were further grouped into nine broad classes: blood, connective, epithelial, germ, immune, muscle, nervous, sensory, and stem (see ShortAnnotations in **Extended Data Table 1** ).

**Cluster Filtering:** Clusters containing fewer than 100 male nuclei were excluded to avoid unreliable *RoX* statistics. This resulted in 385,099 nuclei distributed across 409 distinct cell type clusters.

**Normalization of Expression:** The raw UMI counts for all genes in each remaining nucleus were normalized by dividing by the total UMI count across all genes in the cell, then multiplying by 10,000 using the scanpy function sc.pp.normalize_total with the parameter target_sum=1e4, followed by log transformation using the scanpy function sc.pp.log1p.

**Computing X/A Ratios:** For each nucleus, chromosomes were grouped into two categories: the X chromosome and the autosomes (2L, 2R, 3L, and 3R). The average expression for genes in each group was computed by summing the expression values of all genes on the chromosomes in the group and dividing by the number of non-zero expressed genes. The X/A ratio was then calculated as the ratio of the average expression of the X chromosome to that of the autosomes.

**Computing Dosage Compensation Levels:** For each cluster, male and female cells were analyzed separately, and the median X/A ratio was calculated for each sex, denoted as M (for males) and F (for females). These median values represent the cluster's sex-specific dosage compensation levels. As we consider only male cells in our analysis we use only M, except for the regression analysis (see below), where we used F as one of the predictors of dosage compensation in male cells. A dosage compensation value close to 1 indicates effective compensation. Dosage compensation levels were categorized into three groups based on two thresholds (0.5 and 1.0): anti-compensation (<0.5), effective compensation (0.5–1.0), and overcompensation (>1.0). Clusters were ranked in ascending order of dosage compensation levels.

**Computing and Plotting *RoX* Metrics:** For each cluster, the total *RoX* expression was computed as the sum of the expressions of *RoX1* and *RoX2* . Box plots were generated to visualize these metrics. Median values for all cells within each of the three dosage compensation categories were calculated and represented as horizontal lines on the plots. Statistical significance (p-values) was determined using a t-test, and effect sizes (d) were calculated with Cohen's d function. Both p-values and effect sizes between two groups of clusters were derived using the median values of the clusters.

**Correlations Among *RoX* Expressions and Dosage Compensation:** Pearson partial and total correlations were calculated using the pcor and cor functions in R.

**Scatter Plot of Cluster Medians for RoX1 and RoX2:** Scatter plots were created with dots representing cluster medians of *RoX1* and *RoX2* . Dot colors corresponded to cluster dosage categories (anti-, effective, or over compensation). Ellipses for each group were overlaid using the stat_ellipse function from the ggplot2 package.

**Regression-Based Prediction of Dosage Compensation:** To predict dosage compensation in the male cells, 15 regression models were developed using combinations of the following four independent variables: 1) *RoX1* , 2) *RoX2* , 3) cluster label, and 4) female X/A ratio F, used as a proxy for cluster-level baseline dosage compensation. Cells within male-specific clusters were excluded from the regression analysis as F is undefined for these clusters. Ridge regression was applied to combinations involving discrete cluster label variable, while linear regression was used for the remaining combinations. The adjusted R-squared metrics for each combination were visualized using box plots generated through 10-fold cross-validation, using data from 168,217 male nuclei out of total 385,099 nuclei.

**Code and data availability:** The nuclei level data was downloaded from FCA website (https://flycellatlas.org/; detailed in FCA_Samples, **Extended Data Table 1** ). The code for our analysis and the results are stored in the Zenodo repository 14577924 (https://doi.org/10.5281/zenodo.14577924).

## Reagents

Python packages: loompy, anndata and scanpy for single-nucleus data analysis and scikit-learn for regression analysis. R packages: ggplot for visualization.

## Data Availability

Description: This MS-Excel workbook contains spreadsheets detailing the FCA datasets, clustering resolutions, and the results of our analysis at both the cell and cluster levels. Resource Type: Dataset. DOI: https://doi.org/10.22002/t15w6-x9q23
